# Mechanostat-Informed Strain Mapping of Osseodensification-Inspired Peri-Implant Densification Versus Conventional Drilling in Osteoporotic-like Low-Density Cancellous Bone: A 3D Static Linear Finite Element Analysis

**DOI:** 10.3390/jfb17030149

**Published:** 2026-03-18

**Authors:** Mesut Tuzlali, Nagehan Baki, Nazik İrem Önügören, Kübra Aral, Erkan Bahçe, Cüneyt Asım Aral

**Affiliations:** 1 Department of Prosthodontics, Faculty of Dentistry, Inonu University, 44280 Malatya, Türkiye; nagehan.baki@inonu.edu.tr (N.B.); irem.onugoren@inonu.edu.tr (N.İ.Ö.); 2 Department of Periodontics, Faculty of Dentistry, Inonu University, 44280 Malatya, Türkiye; kubra.aral@inonu.edu.tr (K.A.); cuneyt.aral@inonu.edu.tr (C.A.A.); 3 Department of Mechanical Engineering, Faculty of Engineering, Inonu University, 44280 Malatya, Türkiye; erkan.bahce@inonu.edu.tr

**Keywords:** dental implants, osteoporosis, bone density, finite element analysis, biomechanical, mandible

## Abstract

Low-density cancellous bone results in reduced trabecular support and may increase crestal cortical strain around implants. Osseodensification (OD) compacts trabecular bone and may create a peri-osteotomy densified zone, but its strain-level effects in osteoporotic-like bone are unclear. This study evaluated whether an OD-inspired peri-implant densified trabecular zone reduces crestal cortical strain compared with conventional drilling (CD) in an osteoporotic-like model. A three-dimensional finite element model of a mandibular posterior segment with a 2.0-mm cortical shell and D4 cancellous core was constructed with a 4.3 × 11.4-mm titanium implant and a cemented monolithic zirconia crown. CD used a 4.0-mm osteotomy in D4 bone. The OD model used the same osteotomy plus a concentric peri-implant densified shell with radial density gradation from D1 to D3. The implant–bone interface was defined as bonded. Static 100 N axial and 45° oblique loads were applied. Outcomes were ε_eq_, ε_max_, and ε_min_, summarized as mean top-10 nodal values. OD reduced crestal cortical strains under both loads. Under axial loading, ε_eq_, ε_max_, and |ε_min_| decreased by 17.7%, 19.0%, and 24.1%, respectively. Under oblique loading, the corresponding reductions were 9.8%, 8.0%, and 8.9%. Oblique loading produced higher cortical strains than axial loading in both models. OD-inspired peri-implant densification reduced crestal cortical strain in this osteoporotic-like model, whereas oblique loading remained the main driver of elevated strain. These findings support occlusal/prosthetic strategies that minimize oblique forces and warrant experimental and clinical validation.

## 1. Introduction

Bone-anchored devices are widely used across medicine, ranging from fracture fixation and spine instrumentation to craniofacial reconstruction and dental implant rehabilitation [1]. Despite differences in anatomy and loading conditions, their success depends on stable load transfer at the bone–implant interface. In low-density cancellous (trabecular) bone, reduced structural support may increase local deformation and interfacial micromotion, thereby predisposing fixation systems to loosening or failure, as recognized in osteoporotic bone [2,3].

Osteoporosis is a prevalent systemic skeletal disease and a leading contributor to fragility fractures in adults over 50 years of age [4]. With demographic aging, osteoporosis, osteopenia, and related pharmacotherapy are increasingly encountered in patients seeking implant-based oral rehabilitation [5,6]. Current systematic reviews generally report no consistent reduction in dental implant survival attributable to osteoporosis alone, although modest differences in peri-implant marginal bone level changes have been reported and the certainty of evidence remains limited [7,8]. In addition, clinical risk assessment is also influenced by antiresorptive medications and their association with medication-related osteonecrosis of the jaw (MRONJ). Together, these factors highlight the importance of understanding how low-density cancellous bone may alter the local mechanical environment around implants [9,10,11].

In implant dentistry, low-density cancellous bone is a recognized risk factor for reduced primary stability and unfavorable early mechanical conditions, with more recent evidence indicating that bone quality and cortical support influence implant stability and that type IV bone is associated with a greater risk of early implant failure [12,13]. Osseodensification (OD) was introduced as an alternative osteotomy preparation concept that compacts cancellous bone rather than removing it, with the aim of increasing peri-implant bone density and mechanical engagement. Experimental studies have reported improvements in primary stability, peri-implant bone mineral density, and bone-to-implant contact when OD is compared with conventional subtractive drilling (CD) [14,15,16]. More recently, a systematic review and meta-analysis focusing on low-density bone reported higher primary and secondary stability outcomes with OD compared with CD [17], and a randomized controlled clinical trial also found favorable effects on implant stability and marginal bone levels [18].

From a mechanobiological perspective, bone is a mechanosensitive tissue that adapts to the prevailing mechanical environment. Frost’s mechanostat framework provides a useful conceptual basis for relating tissue-level mechanical strain to bone modeling and remodeling responses, and it has been discussed in relation to osteopenia and osteoporosis [19,20,21]. Within this context, strain-based measures are relevant because they reflect the local mechanical stimulus acting on cortical and cancellous bone adjacent to an implant.

Although OD has been investigated experimentally and clinically, most studies have focused on stability-related outcomes rather than peri-implant strain patterns under functional loading. As a result, the strain-level implications of an OD-associated densified peri-implant zone in osteoporotic-like low-density cancellous bone remain insufficiently characterized [14,15,22]. A strain mapping approach may help clarify whether peri-implant densification alters cortical entry-region strain concentrations and cancellous peri-implant strain patterns under different loading directions [23].

Finite element analysis is increasingly used in dental implant biomechanics to investigate load transfer and to compare protocols and designs, highlighting the importance of standardized modeling assumptions and transparent reporting [24]. Therefore, the aim of this study was to compare peri-implant strain behavior in osteoporotic-like low-density cancellous bone between CD and an OD-inspired peri-osteotomy densification model using three-dimensional finite element analysis. Particular emphasis was placed on high-tail crestal cortical strain responses under axial and 45° oblique loading.

## 2. Materials and Methods

### 2.1. Model Overview

A three-dimensional finite element model was developed to investigate how an osseodensification-inspired peri-implant densified bone layer influences local strain distributions in a low-density (osteoporotic-like) cancellous mandibular segment under static loading. Two osteotomy preparation conditions were evaluated: conventional drilling (CD) and osseodensification (OD). All modeling parameters were kept identical between conditions, except for the presence of the densified peri-implant zone and the minor cortical expansion described below.

### 2.2. Bone Segment Geometry

A posterior mandibular segment corresponding to the first molar region was created in SolidWorks 2024 (Dassault Systèmes, Vélizy-Villacoublay, France). The segment was idealized as a rectangular block measuring 20 mm (mesiodistal) × 8 mm (buccolingual) × 22 mm (vertical). A uniform 2.0-mm cortical shell surrounded a cancellous core. Gingival soft tissue was not included in order to reduce computational complexity and to focus the analysis on peri-implant bone mechanics. To avoid an artificially continuous cortical shell at the segment boundaries, the mesial and distal cut faces were modeled without cortical coverage while preserving continuity of the cancellous core.

### 2.3. Osteotomy Conditions

Two primary osteotomy preparation conditions were defined: conventional drilling (CD) and osseodensification (OD). In the present study, the OD condition was implemented as a combined modeling surrogate that included both a graded peri-osteotomy densified trabecular shell and minor cortical expansion. Accordingly, the analyses were designed to quantify the net OD–CD effect rather than to isolate the individual mechanical contributions of densification and cortical expansion.

**Figure 1:**
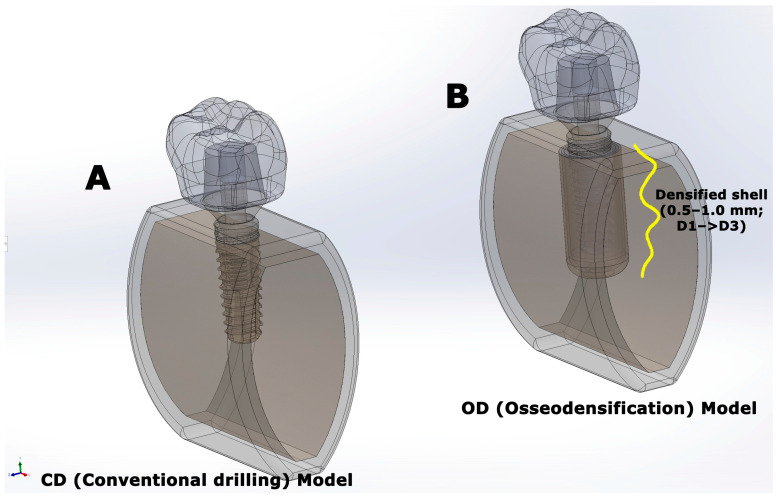
Finite element model geometries used in the analyses. (**A**) Conventional drilling (CD) model and (**B**) osseodensification (OD) model showing the implant–abutment–crown assembly embedded in the bone block (cortical and cancellous bone). The models differed in the osteotomy preparation protocol (CD vs. OD) while all other geometric and material parameters were kept identical.

In the CD condition (Figure 1A), a cylindrical osteotomy with a diameter of 4.0 mm was created and centered within the trabecular compartment. The cancellous bone was assigned low-density (D4) properties to represent a low-modulus trabecular substrate, whereas the cortical shell geometry and material properties were kept identical across all models.

In the OD condition (Figure 1B), the osteotomy diameter was also maintained at 4.0 mm. To represent OD-related compaction, a concentric circumferential densified trabecular shell was added along the osteotomy wall, consistent with histologic descriptions [14,15]. The shell thickness varied axially to mimic the reported gradient along the osteotomy, measuring 0.5 mm laterally and 1.0 mm apically [14,15]. In both models, the native cancellous core was assigned D4 properties to represent osteoporotic-like low-density cancellous bone. In the OD model, the peri-osteotomy shell was graded radially from D1 to D3, with three equal-thickness cancellous zones arranged from the osteotomy wall outward as D1, D2, and D3. Thus, the thickness of each zone was 0.5/3 mm laterally and 1.0/3 mm apically while preserving the same zonal order throughout the shell. In this context, the term “osteoporotic-like” refers to the low-modulus trabecular compartment (D4), whereas the cortical shell material properties were kept constant across models to preserve a controlled OD–CD comparison. In addition, a 0.2 mm buccolingual cortical expansion was incorporated in the OD model, increasing the external buccolingual dimension from 8.0 mm to 8.2 mm, in line with localized outward deformation reported during OD despite the limited intrinsic compressibility of cortical bone [14].

Finally, the OD configuration was intentionally designed as an osseodensification-inspired surrogate intended to represent the reported peri-osteotomy densified layer (graded densified shell), rather than to reproduce a proprietary drill macro-geometry or drilling kinematics. Accordingly, the OD parameters should be interpreted as a standardized modeling surrogate based on histologic and micro-computed tomography (micro-CT) descriptions, and not as a universal “standard OD protocol”.

### 2.4. Implant–Restoration Assembly

A titanium dental implant (4.3 mm × 11.40 mm) with a simplified threaded cylindrical body and internal hex connection was modeled and positioned centrally within the bone segment, equidistant from the buccal and lingual cortical plates. Implant dimensions were selected as a representative posterior implant size to support standardized OD–CD comparisons focused on peri-implant strain responses. A straight titanium abutment with a 6.5-mm diameter and a 3.0-mm transmucosal height was connected to the implant via a modeled abutment screw reproducing the internal thread engagement geometry. A monolithic zirconia crown with mandibular first molar morphology was digitally designed and cemented to the abutment to approximate clinical load transfer through a definitive posterior restoration.

### 2.5. Material Properties

All materials were modeled as homogeneous, isotropic, and linearly elastic, as commonly adopted in implant finite element studies for comparative mechanical analyses [25,26]. Because the primary aim was to isolate the relative mechanical effect of OD versus CD under identical modeling assumptions, this constitutive idealization was adopted for controlled comparative inference rather than absolute physiologic prediction. Material properties for titanium alloy (Ti-6Al-4V), zirconia, cortical bone, and cancellous bone were assigned according to the literature [27,28] and are summarized in Table 1. In the OD model, the densified trabecular layer was assigned a higher Young’s modulus than the native trabecular core, consistent with micro-CT and histomorphometric evidence indicating increased peri-osteotomy mineral density and compaction after OD [14,29]. Material density was not included because the present simulations were linear static; thus, mass and inertia effects were not modeled, and density did not enter the stiffness-based solution for strains under quasi-static loading.

### 2.6. Meshing and Convergence

The assemblies were imported into ANSYS Workbench 2024 R2 (ANSYS Inc., Canonsburg, PA, USA). A tetrahedral mesh was generated with a global element size of 0.1-mm and local refinement at the implant–crestal cortical region, where higher strain gradients were anticipated. Progressive mesh refinement, achieved by reducing element size and thereby increasing the mesh density, is generally expected to improve numerical accuracy and support convergence toward a stable solution, albeit at increased computational cost [30]. Mesh convergence was evaluated using three progressively refined global element sizes (0.5, 0.3, and 0.1 mm). Convergence was considered acceptable when the change in the peri-implant strain outcomes between successive refinements was ≤5%, and the 0.1-mm mesh was therefore selected for all simulations (Supplementary Table S1). The final CD and OD models consisted of approximately 2,333,784/1,321,442 and 3,504,209/1,993,628 nodes and elements, respectively (Supplementary Table S2).

### 2.7. Contact Definitions and Boundary Conditions

Continuity between cortical and cancellous bone, and between densified and native cancellous bone in OD models, was enforced using shared nodes to preserve geometric and material continuity.

The implant–bone interface, including both cortical and trabecular regions, was modeled as perfectly bonded to represent a mature, fully osseointegrated (secondary stability) condition and to isolate OD–CD differences in post-integration load transfer under identical modeling assumptions. The present study was not intended to simulate the immediate post-placement healing phase, during which partial integration, frictional contact, and micromotion govern primary stability [25].

For the two-piece implant configuration, the implant–abutment and abutment–screw interfaces were also defined as bonded. The crown–abutment interface was likewise modeled as bonded to represent a cemented connection. Screw preload was not simulated; interfaces were idealized as bonded to isolate the effect of peri-implant densification.

**Figure 2:**
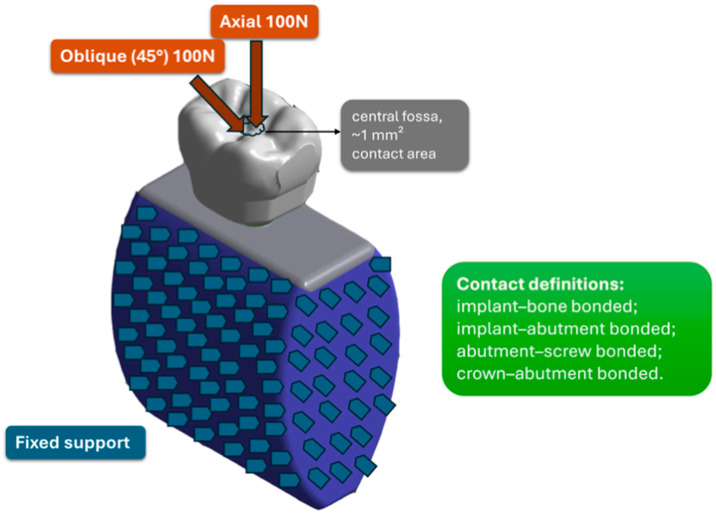
Schematic representation of the finite element model setup, illustrating axial and 45° oblique loading, fixed boundary constraints, and bonded contact definitions used in the analyses.

To prevent rigid body motion and approximate fixation of the mandibular segment, the mesial and distal ends of the bone block were fully constrained in all degrees of freedom. The inferior surface was also fixed, providing global stabilization during load application. This constraint scheme was selected to suppress rigid body motion and ensure consistent boundary conditions across models; therefore, outcomes were interpreted comparatively (OD and CD) rather than as absolute physiologic magnitudes (Figure 2).

### 2.8. Loading Conditions

Two static loading configurations were simulated to represent posterior occlusal loading. For axial loading, a 100 N vertical force was applied perpendicular to the occlusal surface at the central fossa and distributed over an approximately 1 mm^2^ contact area. For oblique loading, a 100 N force was applied at 45° to the implant’s long axis at the same central fossa contact area and resolved into orthogonal components along the global coordinate axes (Figure 2).

The applied load magnitude (100 N) was selected as a standardized comparative probe load, consistent with ranges commonly used in implant biomechanics and dental FEA studies, to enable controlled OD–CD comparisons while avoiding unrealistically high local strain spikes in low-density trabecular substrates. Oblique loading at 45° was included because off-axis occlusal contacts generate larger bending moments and are commonly used to represent clinically relevant non-axial functional loading components that amplify crestal cortical strain patterns [31,32,33]. Accordingly, axial and 45° oblique cases were used to bracket a representative spectrum of loading directions under identical boundary and contact assumptions, and outcomes were interpreted primarily in terms of OD–CD relative differences rather than absolute patient-specific physiologic magnitudes.

### 2.9. Outcome Measures and Strain Post-Processing

Primary outcomes were strain-based metrics in the peri-implant cortical region, including equivalent (von Mises) elastic strain (ε_eq_), maximum principal elastic strain (ε_max_; tensile), and minimum principal elastic strain (ε_min_; compressive).

To reduce the influence of localized numerical spikes arising from geometric transitions, contact edges, or mesh-related singularities, results were not interpreted using single-point maxima [34,35]. Instead, strain data were exported from ANSYS Workbench and processed externally. For each region of interest, a high-tail descriptor was calculated as the mean value of the 10 nodes exhibiting the highest strain magnitudes, thereby mitigating the effect of numerical artifacts while preserving sensitivity to peak mechanical demand. For compressive strain, comparisons were based on the absolute magnitude of ε_min_. Post-processing was performed using Microsoft Excel (Microsoft Corp., Redmond, WA, USA).

All strain values were reported as dimensionless strain (mm/mm) and additionally expressed in microstrain (µε) for interpretability (µε = strain × 10^6^). Comparative evaluation between CD and OD models was performed by examining the magnitude and spatial distribution of strain fields under axial and oblique loading, supported by contour maps.

## 3. Results

The results are presented as comparative cortical strain outcomes for the CD and OD models under axial and oblique loading. Detailed findings are reported by strain metric in the following subsections.

### 3.1. Cortical Bone Strain Outcomes

**Figure 3:**
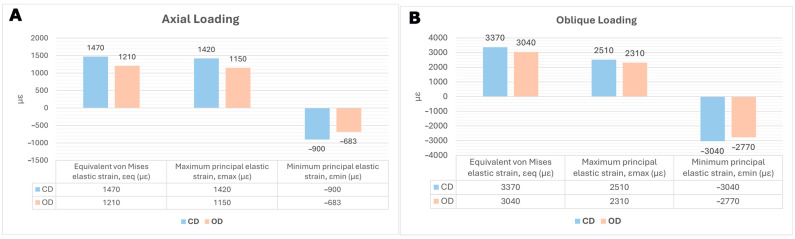
Mean peak peri-implant cortical bone elastic strain under (**A**) axial and (**B**) oblique loading for the CD and OD models. Values are reported in microstrain (µε); negative values indicate compressive strain. For each outcome, the mean of the 10 nodes representing the high-tail response (as defined in Table 2) was computed.

**Figure 4:**
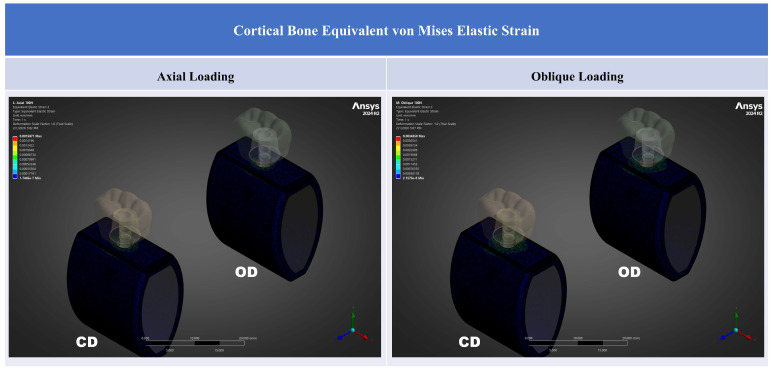
Cortical bone equivalent (von Mises) elastic strain (ε_eq_) contours for CD and OD models under axial and oblique loading. Contours are displayed in mm/mm (1 mm/mm = 10^6^ µε).

**Figure 5:**
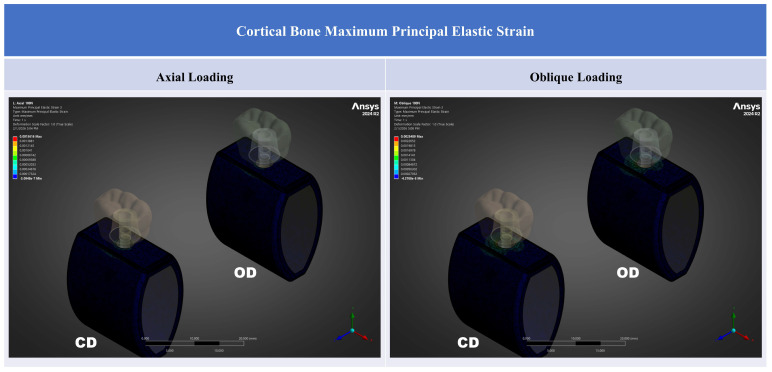
Cortical bone maximum principal elastic strain (ε_max_) contours for CD and OD models under axial loading and oblique loading. Contours are displayed in mm/mm (1 mm/mm = 10^6^ µε).

**Figure 6:**
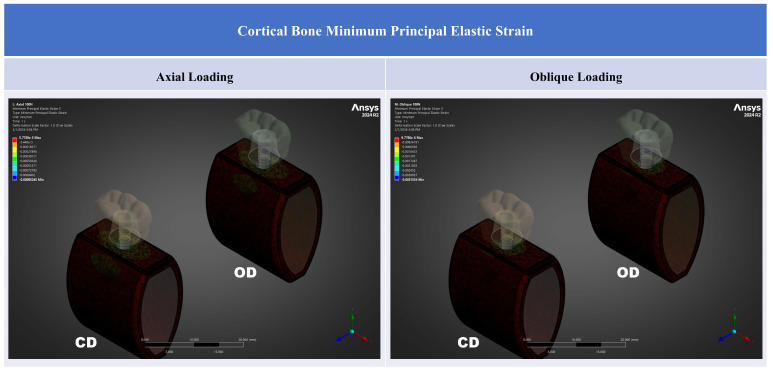
Cortical bone minimum principal elastic strain (ε_min_) contours for CD and OD models under axial loading and oblique loading. Contours are displayed in mm/mm (1 mm/mm = 10^6^ µε); negative values indicate compressive strain.

Peri-implant cortical bone strain was consistently lower in the OD model than in the CD model under both loading directions (Table 2; Figure 3, Figure 4, Figure 5 and Figure 6). Strain concentrations were located primarily in the crestal cortical collar surrounding the implant neck, as demonstrated by the contour maps (Figure 4, Figure 5 and Figure 6). Values are reported in microstrain (µε), where 1 µε = 10^−6^ strain (mm/mm).

Across all strain metrics, the OD condition showed a consistent reduction in high-tail crestal cortical strain relative to CD (Table 2). Oblique loading markedly amplified cortical strains compared with axial loading in both models (Figure 3). For ε_eq_, oblique-to-axial amplification was approximately 2.29-fold in CD (3370/1470) and 2.51-fold in OD (3040/1210). For ε_max_, the corresponding amplification was approximately 1.77-fold in CD (2510/1420) and 2.01-fold in OD (2310/1150). For compressive strain magnitude (|ε_min_|), amplification was approximately 3.38-fold in CD (3040/900) and 4.06-fold in OD (2770/683). Thus, although OD reduced cortical strain under both loading directions, oblique loading produced substantially higher crestal cortical strain than axial loading in both models.

Contour plots show that the highest strain regions were concentrated at the crestal cortical collar adjacent to the implant neck, consistent with a bending-dominant load transfer pattern under posterior loading. Under oblique loading, both the magnitude and spatial extent of high-strain regions increased for all strain descriptors, whereas the OD model showed a visibly attenuated high-strain field compared with the CD model within the crestal cortical region (Figure 4, Figure 5 and Figure 6).

### 3.2. Equivalent (von Mises) Elastic Strain

Under axial loading (100 N), the mean high-tail ε_eq_ decreased from 1470 µε in CD model to 1210 µε in OD (Δ = −260 µε; −17.7%). Under oblique loading (100 N at 45°), ε_eq_ decreased from 3370 µε in CD to 3040 µε in OD (Δ = −330 µε; −9.8%) (Figure 4). The relative reduction was greater under axial loading than under oblique loading. The ε_eq_ contour maps showed that OD reduced the intensity of the crestal cortical high-strain zone under both loading directions, with the greater attenuation observed under axial loading.

### 3.3. Maximum Principal (Tensile) Elastic Strain

For axial loading, ε_max_ decreased from 1420 µε in the CD model to 1150 µε in the OD model (Δ = −270 µε; −19.0%). For oblique loading, ε_max_ decreased from 2510 µε in CD to 2310 µε in OD (Δ = −200 µε; −8.0%) (Figure 5). As with ε_eq_, the relative reduction was greater under axial loading, whereas oblique loading produced higher tensile strain levels in both models (Table 2). Tensile strain hotspots were concentrated at the crestal cortical collar and increased under oblique loading; OD consistently reduced the peak tensile strain concentration relative to CD.

### 3.4. Minimum Principal (Compressive) Elastic Strain

Compressive strain magnitudes were lower in the OD model under both loading directions. Under axial loading, ε_min_ changed from −900 µε in CD to −683 µε in OD, corresponding to a 24.1% reduction in compressive strain magnitude (|ε_min_|). Under oblique loading, ε_min_ changed from −3040 µε in CD to −2770 µε in OD, corresponding to an 8.9% reduction in |ε_min_| (Figure 6). Among the reported descriptors, the largest relative OD-related effect was observed for axial compressive strain magnitude, whereas under oblique loading the reductions remained directionally consistent but smaller (Table 2). Compressive strain peaks were localized at the crestal cortical collar and increased markedly under oblique loading; OD reduced compressive strain magnitude (|ε_min_|) under both loading directions.

## 4. Discussion

This finite element analysis investigated whether an osseodensification (OD)-inspired peri-osteotomy densified trabecular zone can meaningfully alter peri-implant strain demand in a low-stiffness (“osteoporotic-like”) cancellous environment. Under both axial and 45° oblique loading, the OD model consistently reduced high-tail strain metrics in the crestal cortical region compared with CD, with a larger relative effect under axial loading (~18–24% depending on metric) and a smaller but directionally consistent effect under oblique loading (~8–10%). These findings support the biomechanical plausibility that locally increasing peri-implant trabecular stiffness—conceptually consistent with OD-associated densification—can partially offload the crestal cortical collar, a region widely recognized as a mechanical hotspot under bending-dominant loading [19,25].

### 4.1. Mechanostat-Informed Interpretation of Peri-Implant Strain Magnitudes

Interpreting peri-implant strain within the framework of bone mechanoregulation is useful when strain is regarded as a relative indicator of mechanical stimulus rather than a direct surrogate for clinical failure. Frost’s mechanostat framework proposes broad strain ranges within which bone tends to maintain, model, or remodel, with modeling activity becoming more likely as strain rises into approximately the 1500–3000 µε range in vivo, although such thresholds remain conceptual and context-dependent. Commonly cited mechanostat ranges include disuse (≤~50–100 µε), adapted window (~1000–1500 µε), overload (~1500–3000 µε), and pathological overload (≥~3000 µε); whereas fracture or failure has generally been associated with substantially higher strain levels, often ≥~15,000 µε [19,36].

In the present model, oblique loading shifted the crestal cortical region into a higher strain regime than axial loading, consistent with the expectation that lateral components generate bending moments and concentrate deformation cervically. Within this context, the OD-inspired densification shifted high-tail crestal cortical strains downward under both load directions, suggesting a mechanical environment less likely to operate in a high-strain upper-tail condition. This is particularly relevant when bone quality is reduced and trabecular load sharing is diminished.

Experimental mechanobiology further indicates that skeletal adaptation is influenced not only by strain magnitude but also by stimulus characteristics such as cycle structure and rest insertion, and that adaptive responses can behave in an approximately monotonic or near-linear manner across broad ranges between disuse and high-strain conditions [20]. In addition, FEA-based bone adaptation frameworks have shown that predicted peri-implant adaptation can be sensitive to the selected mechanical stimulus definition and modeling parameters, reinforcing that strain metrics are most appropriately interpreted as relative indicators within a standardized modeling context [37].

Accordingly, the present results should be interpreted as evidence of a favorable redistribution of strain demand at the crestal cortical region rather than as a direct predictor of marginal bone loss or of exceeding a definitive “safe” or “unsafe” threshold.

### 4.2. Relationship to the Osseodensification Evidence Base

OD has primarily been positioned as a site-preparation approach intended to preserve and compact bone, thereby creating a densified layer at the osteotomy wall and improving mechanical engagement. This conceptual mechanism is supported by preclinical and clinical observations. Histologic and micro-CT-based studies have described peri-osteotomy densification patterns and improved stability-related outcomes consistent with this mechanism of OD [14,15,38].

Clinical evidence also supports improved stability-related outcomes with OD. A multicenter controlled trial reported higher insertion torque and favorable temporal ISQ behavior with OD relative to subtractive drilling [16], and a more recent randomized controlled clinical trial reported effects on implant stability and marginal bone levels compared with CD [18]. Systematic reviews and meta-analyses likewise report improvements in primary, and in some analyses secondary, stability surrogates with OD compared with CD in low-density bone while emphasizing heterogeneity in protocols and outcomes, and the limited availability of long-term hard-endpoint clinical evidence [17,22].

Within this evidence base, the present study adds a complementary strain-based perspective. Whereas most OD studies have focused on insertion torque, ISQ, or marginal bone level changes, the present analysis quantified how an OD-inspired densified peri-osteotomy zone may redistribute functional strain demand in the crestal cortical collar under standardized loading. Mechanically, increasing peri-implant trabecular stiffness improves load sharing around the osteotomy wall and may reduce the concentration of deformation at the cortical crest, particularly under predominantly axial loading. The smaller effect under observed under oblique loading is also mechanically intuitive: bending moments amplify crestal deformation even when trabecular support is stiffened, implying that densification may not fully mitigate off-axis overload.

### 4.3. Comparison with Previous Finite Element Evidence

Recent bibliometric evidence highlights the rapid growth and diversification of dental biomechanics FEA, reinforcing the need for a clear definition of modeling assumptions and comparative interpretation when placing new simulations within the broader evidence base [24].

The pronounced load-direction dependence observed in this study is consistent with previous implant FEA literature showing that oblique loading (e.g., 45°) increases deformation and elevates peri-implant cortical strain demand compared with axial loading, reflecting bending-dominant load transfer at the crestal collar [31,39]. A broader systematic perspective further indicates that implant FEA studies frequently evaluate oblique forces while often simplifying bone as isotropic and omitting muscle forces and remodeling, supporting the use of standardized boundary and loading conditions for controlled comparisons [25]. Taken together, these trends support interpretation of the present OD-CD differences as stiffness-driven modulation of crestal collar demand under bending-sensitive loading.

In addition, FEA studies that vary bone condition, density, and osseointegration assumptions generally report that reduced trabecular support shifts load sharing toward the cortical shell, thereby accentuating crestal concentration patterns under functional loading directions [25,40]. Within this broader framework, the OD-inspired parameterization used here, namely graded peri-osteotomy densification, produced a consistent reduction in high-tail crestal cortical strain relative to CD. This finding is mechanically coherent with the premise that increased peri-osteotomy stiffness improves local load sharing and attenuates cortical collar extremes [41,42].

Similarly, numerical peri-implant bone adaptation studies comparing alternative mechanical stimuli have shown that stimulus choice and model parameterization can influence predicted adaptation patterns, supporting the use of standardized comparative setups when interpreting differences in strain fields [37]. OD-specific FEA modeling remains comparatively limited, as most OD evidence has focused on insertion torque, ISQ, and histologic or imaging-based descriptions of densification. The present study therefore provides complementary strain-field insight by quantifying direction-dependent high-tail cortical responses under standardized loading [25,34].

### 4.4. Why the Crestal Cortical Region Is a Critical Area in Low-Density Cancellous Bone

When cancellous stiffness is reduced, the cortical shell may bear a disproportionate share of load, thereby increasing local strain gradients at the crestal collar under functional loading. This concept is consistent with broader orthopedic discussions of fixation mechanics in osteoporotic bone, in which reduced bone quality can amplify interface deformation demands [2,3].

In implant dentistry, this issue also intersects with immediate and early loading biomechanics, where excessive implant–bone micromotion has been implicated in impaired osseointegration, and stable mechanical conditions are considered prerequisites for predictable osseointegration [43,44].

Although the present simulations assumed a perfectly bonded (osseointegrated) interface, the observed reduction in high-tail cortical region strains in the OD model supports the hypothesis that a densified peri-osteotomy zone may provide a mechanical buffer in compromised cancellous environments while still leaving oblique loading as the dominant driver of peak crestal strain demand.

### 4.5. Clinical Implications and Cautious Translation

Clinically, OD is often selected in low-quality bone to enhance primary stability. The present findings provide a plausible mechanical complement to this rationale: if peri-osteotomy densification increases effective local stiffness, crestal cortical strain concentrations under functional loads may be reduced, potentially moderating mechanically driven remodeling stimuli at the cortical collar [16,19].

However, translation should remain cautious. A static, linear-elastic, fully bonded model does not account for time-dependent remodeling, viscoelasticity, partial integration, or cyclic fatigue. Moreover, posterior bite forces can substantially exceed 100 N depending on patient-specific factors and measurement conditions; therefore, the present loads should be interpreted as standardized comparative probes rather than as patient-specific physiologic maxima [45].

From a clinical biomechanics standpoint, the strong load-direction dependence observed here reinforces that occlusal and prosthetic strategies aimed at reducing off-axis forces such as occlusal scheme optimization, control of cusp guidance, cantilever management, and appropriate stiffness or splinting considerations, remain fundamental adjuncts to any osteotomy-based approach.

### 4.6. Limitations and Future Research Directions

While the present findings support a mechanically favorable OD-associated redistribution of crestal cortical strain under standardized conditions, several study-specific limitations should be considered when interpreting the magnitude, mechanism, and clinical transferability of these effects. First, bone was modeled as homogeneous, isotropic, and linearly elastic. Although this simplification is common in comparative implant FEA, it does not capture trabecular heterogeneity, anisotropy, or time-dependent behavior such as viscoelasticity. As a result, the absolute magnitude of reported strains, particularly in the high-strain tail, may differ from those expected under more physiologic conditions, especially during sustained or cyclic loading [25,46,47]. Accordingly, the present OD–CD differences should be interpreted primarily as relative comparative trends rather than patient-specific physiologic predictions. In addition, a standardized posterior mandibular segment with fully constrained mesial and distal ends and inferior surface was used to suppress rigid-body motion and ensure internally consistent OD–CD comparisons. However, this idealized setup does not reproduce full-mandible anatomy, muscle forces, or physiologic support conditions. Because boundary conditions can influence strain transfer patterns, they may also affect the apparent magnitude of crestal cortical demand under both axial and oblique loading. Future studies should therefore incorporate CT-based heterogeneous mapping and, where feasible, anisotropic and time-dependent constitutive formulations, while also moving toward CT-derived mandibular geometries with more anatomically realistic support conditions and muscle-force-informed loading strategies.

A further limitation concerns the interface assumptions adopted in the present model. The implant–bone interface was modeled as perfectly bonded to represent mature osseointegration and post-integration load transfer. Although this assumption is appropriate for comparative evaluation of secondary stability conditions, it does not capture early healing phases in which frictional contact, partial integration, and micromotion govern primary stability [43,44]. Therefore, the present findings should not be interpreted as direct evidence regarding immediate or early loading stability. In addition, the OD condition was implemented as a combined surrogate consisting of a graded densified peri-osteotomy shell together with minor cortical expansion. Consequently, the present model supports inference on the net OD-associated mechanical effect, but it does not isolate whether the observed reduction in crestal cortical strain was driven predominantly by trabecular densification, cortical expansion, or the interaction between these components. This limits mechanistic attribution even though the overall OD–CD comparison remains informative. Future work should therefore compare bonded and frictional/contact-based interface conditions and introduce factor-isolation models, including densification-only, expansion-only, and combined configurations, in order to quantify the main effects and interactions more explicitly.

The OD parameterization itself also represents a standardized idealization. The thickness of the densified zone (0.5–1.0 mm), its radial grading (D1→D3), and the associated modulus assignments were prescribed to reflect histologic and imaging-supported descriptions of OD-associated densification. Nevertheless, the extent and intensity of peri-osteotomy densification are likely to vary across patients, bone qualities, implant sites, and drilling protocols. Thus, the effect sizes observed here should be understood as representative of a standardized OD-inspired surrogate rather than a universal estimate applicable to all OD scenarios. At the outcome-analysis level, high-tail strain was summarized as the mean of the 10 nodes with the highest strain values within the selected region of interest (ROI) in order to reduce sensitivity to isolated nodal spikes caused by mesh- or geometry-related numerical artifacts. However, because the main conclusions of the study were based on relative reductions in high-tail crestal cortical strain, the choice of a top-10 descriptor may still be perceived as somewhat arbitrary and may depend on ROI node count. In addition, the principal outcome measures were derived from the crestal cortical collar, which was selected because it is a recognized biomechanical hotspot under implant loading and was central to the mechanistic interpretation developed in the present discussion. However, the magnitude of high-tail strain metrics may vary depending on ROI definition and anatomical extent, meaning that the observed OD–CD effect size may not be identical in deeper cancellous regions or other peri-implant zones. Future research should therefore perform systematic parametric sweeps of shell thickness, grading patterns, and modulus ratios; calibrate these parameters against experimental and/or imaging-based densification measurements whenever possible; and report sensitivity analyses using alternative tail definitions and multiple clinically relevant ROIs.

Finally, loading was modeled as a static force applied over a single occlusal contact area. In vivo mastication is cyclic, multi-contact, and history-dependent, and these additional features may alter mechanobiologic adaptation patterns [20]. This limitation is particularly important because the present results demonstrated strong load-direction dependence, with oblique loading remaining the dominant driver of crestal cortical strain demand despite the OD-associated reduction in strain. In addition, although the model provides mechanistic insight into OD-associated strain redistribution, direct validation against experimental strain measurements or calibrated low-density cancellous bone analogs was not performed. Therefore, the present findings should be regarded as mechanistically informative rather than experimentally validated predictions of in vivo peri-implant behavior. Future studies should incorporate cyclic multi-contact loading conditions and, where feasible, couple the mechanical model with mechanobiologic remodeling formulations, while also validating the model against strain-gauge or digital image correlation measurements in bone analogs and/or experimental protocols specifically designed to quantify peri-implant densification effects.

Overall, broader adoption of contemporary in-silico reporting frameworks, including Reporting Guidelines for In Silico Studies using Finite Element Analysis in Medicine (RIFEM), together with implant-FEA best-practice recommendations, would further improve transparency, reproducibility, and interpretability in this field [25,48]. The continued expansion of the dental FEA literature further underscores the importance of standardized reporting and reproducibility practices [24].

## 5. Conclusions

Within the limitations of this idealized, fully osseointegrated finite element model of osteoporotic-like low-density cancellous bone, an OD-inspired graded peri-implant densified trabecular zone consistently reduced the high-tail crestal cortical strain under both axial and 45° oblique loading. The reduction was greater under axial loading than under oblique loading. Oblique loading remained the dominant driver of elevated crestal cortical strain, emphasizing the importance of off-axis load control in low-density cancellous bone even when peri-implant densification is present. These findings support the biomechanical rationale for peri-implant densification as a strategy to reduce crestal cortical deformation, while underscoring the need for experimental validation and standardized, distribution-aware post-processing to improve reproducibility and clinical translation.

## Figures and Tables

**Table 1:** Mechanical properties used in the finite element analyses, including Young’s modulus and Poisson’s ratio for cortical bone, graded cancellous bone (D1–D4), titanium components, and zirconia.

Material	Modulus of Elasticity (GPa)	Poisson’s Ratio (ν)	Reference
Cortical bone	14.8	0.30	[27]
Cancellous bone D1	9.5	0.30	[27]
Cancellous bone D2	5.5	0.30	[27]
Cancellous bone D3	1.6	0.30	[27]
Cancellous bone D4	0.69	0.30	[27]
Titanium (implant, abutment)	110	0.35	[27]
Zirconia	210	0.33	[28]

**Table 2:** Mean high-tail peri-implant cortical bone elastic strain under axial and oblique loading (top-10 node averaging). Values are reported in microstrain (µε). Negative values indicate compressive strain. For ε_eq_ and ε_max_, the mean of the 10 nodes exhibiting the highest strain values within the cortical region was computed. For ε_min_, the mean of the 10 nodes exhibiting the most negative (i.e., most compressive) strain values was computed. Percentage change (Δ%) was calculated as (OD − CD)/CD × 100 for ε_eq_ and ε_max_. For ε_min_, Δ% was calculated using strain magnitudes: (|OD| − |CD|)/|CD| × 100.

Strain Measure (Cortical Bone)	Axial Loading			Oblique Loading		
	CD (µε)	OD (µε)	Δ%	CD (µε)	OD (µε)	Δ%
Equivalent von Mises elastic strain, ε_eq_	1470	1210	−17.7%	3370	3040	−9.8%
Maximum principal elastic strain, ε_max_	1420	1150	−19.0%	2510	2310	−8.0%
Minimum principal elastic strain, ε_min_	−900	−683	−24.1%	−3040	−2770	−8.9%

CD, conventional drilling; OD, osseodensification. For ε_eq_ and ε_max_, high-tail strain was defined as the mean of the 10 highest nodal values; for ε_min_, as the mean of the 10 most compressive nodal values. Percentage change (Δ%) was calculated as described in the Methods.

## Data Availability

The datasets used and/or analyzed during the current study are available from the corresponding author on reasonable request.

## Supplementary Materials

The following supporting information can be downloaded at: https://www.mdpi.com/article/10.3390/jfb17030149/s1, Table S1: Mesh convergence results for high-tail peri-implant strain (Top-10 nodal mean εeq) in the crestal cortical ROI (0.5/0.3/0.1 mm). Table S2: Component-wise mesh statistics (nodes and elements) for CD and OD models.

## Abbreviations

The following abbreviations are used in this manuscript:

CD	Conventional drilling
D1–D4	Cancellous bone density classes (Lekholm & Zarb classification)
FEA	Finite element analysis
ISQ	Implant stability quotient
Micro-CT	Micro-computed tomography
MRONJ	Medication-related osteonecrosis of the jaw
OD	Osseodensification
ROI	Region of interest
ε_eq_	Equivalent (von Mises) elastic strain
ε_max_	Maximum principal (tensile) elastic strain
ε_min_	Minimum principal (compressive) elastic strain
µε	Microstrain (10^−6^ strain)

## References

[1] Isaacson B.M., Jeyapalina S. (2014). Osseointegration: A review of the fundamentals for assuring cementless skeletal fixation. Orthop. Res. Rev..

[2] Mukhopadhaya J., Bhadani J.S. (2025). Fixation Failure in Osteoporotic Bone: A Review of Complications and Outcomes. Indian J. Orthop..

[3] Tandon V., Franke J., Kalidindi K.K.V. (2020). Advancements in osteoporotic spine fixation. J. Clin. Orthop. Trauma.

[4] Sözen T., Özışık L., Başaran N. (2017). An overview and management of osteoporosis. Eur. J. Rheumatol..

[5] Shen Y., Huang X., Wu J., Lin X., Zhou X., Zhu Z., Pan X., Xu J., Qiao J., Zhang T. (2022). The Global Burden of Osteoporosis, Low Bone Mass, and Its Related Fracture in 204 Countries and Territories, 1990–2019. Front. Endocrinol..

[6] Sànchez-Riera L., Wilson N., Kamalaraj N., Nolla J.M., Kok C., Li Y., Macara M., Norman R., Chen J.S., Smith E.U. (2010). Osteoporosis and fragility fractures. Best Pract. Res. Clin. Rheumatol..

[7] de Medeiros F., Kudo G.A.H., Leme B.G., Saraiva P.P., Verri F.R., Honório H.M., Pellizzer E.P., Santiago Junior J.F. (2018). Dental implants in patients with osteoporosis: A systematic review with meta-analysis. Int. J. Oral Maxillofac. Surg..

[8] Lemos C.A.A., de Oliveira A.S., Faé D.S., Oliveira H., Del Rei Daltro Rosa C.D., Bento V.A.A., Verri F.R., Pellizzer E.P. (2023). Do dental implants placed in patients with osteoporosis have higher risks of failure and marginal bone loss compared to those in healthy patients? A systematic review with meta-analysis. Clin. Oral Investig..

[9] Seo D.D., Borke J.L. (2024). Medication-Related Osteonecrosis of the Jaw–2024 Update. Oral Health Dent. Sci..

[10] Andersen S.W.M., Hindocha N.V., Poulsen I., Schliephake H., Jensen S.S. (2025). Medication-Related Osteonecrosis of the Jaws in Patients on Antiresorptive Medication With Dental Implants. A Scoping Review. Clin. Oral Implant. Res..

[11] Ruggiero S.L., Dodson T.B., Aghaloo T., Carlson E.R., Ward B.B., Kademani D. (2022). American Association of Oral and Maxillofacial Surgeons’ Position Paper on Medication-Related Osteonecrosis of the Jaws-2022 Update. J. Oral Maxillofac. Surg..

[12] Rues S., Schmitter M., Kappel S., Sonntag R., Kretzer J.P., Nadorf J. (2021). Effect of bone quality and quantity on the primary stability of dental implants in a simulated bicortical placement. Clin. Oral Investig..

[13] Yari A., Fasih P., Alborzi S., Nikzad H., Romoozi E. (2024). Risk factors associated with early implant failure: A retrospective review. J. Stomatol. Oral Maxillofac. Surg..

[14] Huwais S., Meyer E.G. (2017). A Novel Osseous Densification Approach in Implant Osteotomy Preparation to Increase Biomechanical Primary Stability, Bone Mineral Density, and Bone-to-Implant Contact. Int. J. Oral Maxillofac. Implant..

[15] Trisi P., Berardini M., Falco A., Podaliri Vulpiani M. (2016). New Osseodensification Implant Site Preparation Method to Increase Bone Density in Low-Density Bone: In Vivo Evaluation in Sheep. Implant. Dent..

[16] Bergamo E.T.P., Zahoui A., Barrera R.B., Huwais S., Coelho P.G., Karateew E.D., Bonfante E.A. (2021). Osseodensification effect on implants primary and secondary stability: Multicenter controlled clinical trial. Clin. Implant. Dent. Relat. Res..

[17] Mohammadi M., Mohamadi Moghadam M., Arab-Zozani M. (2025). Primary and secondary stability in implants placed in low-density bone using conventional vs. osseodensification technique: A systematic review and meta-analysis. BMC Oral Health.

[18] Politi I., Honari B., Winning L., Polyzois I. (2025). The Effect of Osseodensification on Implant Stability and Marginal Bone Levels: A Randomized Control Clinical Trial. Clin. Exp. Dent. Res..

[19] Frost H.M. (2003). Bone’s mechanostat: A 2003 update. Anat. Rec. A Discov. Mol. Cell. Evol. Biol..

[20] Sugiyama T., Meakin L.B., Browne W.J., Galea G.L., Price J.S., Lanyon L.E. (2012). Bones’ adaptive response to mechanical loading is essentially linear between the low strains associated with disuse and the high strains associated with the lamellar/woven bone transition. J. Bone Min. Res..

[21] Marques F.C., Boaretti D., Walle M., Scheuren A.C., Schulte F.A., Müller R. (2023). Mechanostat parameters estimated from time-lapsed in vivo micro-computed tomography data of mechanically driven bone adaptation are logarithmically dependent on loading frequency. Front. Bioeng. Biotechnol..

[22] Fontes Pereira J., Costa R., Nunes Vasques M., Salazar F., Mendes J.M., Infante da Câmara M. (2023). Osseodensification: An Alternative to Conventional Osteotomy in Implant Site Preparation: A Systematic Review. J. Clin. Med..

[23] Meslier Q.A., Shefelbine S.J. (2023). Using Finite Element Modeling in Bone Mechanoadaptation. Curr. Osteoporos. Rep..

[24] Xie B., Zhang L., Wang Y., Chu Y., Lu Y. (2025). Finite element analysis in the Dental Sciences: A Bibliometric and a Visual Study. Int. Dent. J..

[25] Falcinelli C., Valente F., Vasta M., Traini T. (2023). Finite element analysis in implant dentistry: State of the art and future directions. Dent. Mater..

[26] Kriswanto K., Jamari J., Andika R., Bayuseno A.P., Yusuf A.A., Ammarullah M.I. (2025). Fixture design effects on posterior dental implant stability using finite element analysis (FEA): A systematic review. Head Face Med..

[27] Premnath K., Sridevi J., Kalavathy N., Nagaranjani P., Sharmila M.R. (2013). Evaluation of stress distribution in bone of different densities using different implant designs: A three-dimensional finite element analysis. J. Indian Prosthodont. Soc..

[28] Fabris D., Souza J.C.M., Silva F.S., Fredel M., Mesquita-Guimarães J., Zhang Y., Henriques B. (2016). The bending stress distribution in bilayered and graded zirconia-based dental ceramics. Ceram. Int..

[29] Lahens B., Neiva R., Tovar N., Alifarag A.M., Jimbo R., Bonfante E.A., Bowers M.M., Cuppini M., Freitas H., Witek L. (2016). Biomechanical and histologic basis of osseodensification drilling for endosteal implant placement in low density bone. An experimental study in sheep. J. Mech. Behav. Biomed. Mater..

[30] Pisarciuc C., Dan I., Cioară R. (2023). The Influence of Mesh Density on the Results Obtained by Finite Element Analysis of Complex Bodies. Materials.

[31] Aunmeungtong W., Khongkhunthian P., Rungsiyakull P. (2016). Stress and strain distribution in three different mini dental implant designs using in implant retained overdenture: A finite element analysis study. Oral Implantol..

[32] Talreja K.S., Rodrigues S.J., Pai U.Y., Shetty T., Saldanha S., Mahesh M., Hegde P., Shenoy S.B., Naik N., Mukherjee S. (2023). A Nonlinear Three-Dimensional Finite Element Analysis of Stress Distribution and Microstrain Evaluation in Short Dental Implants with Three Different Implant-Abutment Connections in Single and Splinted Conditions in the Posterior Mandible. Int. J. Dent..

[33] Sadr K., Vahid Pakdel S.M. (2022). A 3-D finite element analysis of the effect of dental implant thread angle on stress distribution in the surrounding bone. J. Dent. Res. Dent. Clin. Dent. Prospect..

[34] Roffmann O., Stiesch M., Greuling A. (2024). Preventing stress singularities in peri-implant bone—A finite element analysis using a graded bone model. Comput. Methods Biomech. Biomed. Eng..

[35] İlter Er Ö., Çelenk S. (2025). Finite Element Analysis of Stress Distribution in Immature Permanent Incisors Following MTA Apexification with Different Coronal Base Materials. Biomimetics.

[36] Frost H.M. (1987). Bone “mass” and the “mechanostat”: A proposal. Anat. Rec..

[37] Piccinini M., Cugnoni J., Botsis J., Ammann P., Wiskott A. (2016). Numerical prediction of peri-implant bone adaptation: Comparison of mechanical stimuli and sensitivity to modeling parameters. Med. Eng. Phys..

[38] Lahens B., Lopez C.D., Neiva R.F., Bowers M.M., Jimbo R., Bonfante E.A., Morcos J., Witek L., Tovar N., Coelho P.G. (2019). The effect of osseodensification drilling for endosteal implants with different surface treatments: A study in sheep. J. Biomed. Mater. Res. B Appl. Biomater..

[39] Baek Y.-W., Lim Y.-J., Kim B. (2023). Comparison of Implant Surgery Methods of Cortical Tapping and Cortical Widening in Bone of Various Density: A Three-Dimensional Finite Element Study. Materials.

[40] Yang Y., Liu Y., Yuan X., Ren M., Chen X., Luo L., Zheng L., Liu Y. (2023). Three-dimensional finite element analysis of stress distribution on short implants with different bone conditions and osseointegration rates. BMC Oral Health.

[41] González-Mederos P., Rodríguez-Guerra J., González J.E., Picardo A., Torres Y. (2025). A Finite Element Analysis of a New Dental Implant Design: The Influence of the Diameter, Length, and Material of an Implant on Its Biomechanical Behavior. Materials.

[42] Londono J.J., Ramos A., Correa S., Mesnard M. (2020). Influence of dental implant designs on the strain at the peri-implant cortical bone: A finite element study. Comput. Methods Biomech. Biomed. Eng..

[43] Kohli N., Stoddart J.C., van Arkel R.J. (2021). The limit of tolerable micromotion for implant osseointegration: A systematic review. Sci. Rep..

[44] Winter W., Klein D., Karl M. (2013). Micromotion of Dental Implants: Basic Mechanical Considerations. J. Med. Eng..

[45] Koc D., Dogan A., Bek B. (2010). Bite force and influential factors on bite force measurements: A literature review. Eur. J. Dent..

[46] Manda K., Xie S., Wallace R.J., Levrero-Florencio F., Pankaj P. (2016). Linear viscoelasticity-bone volume fraction relationships of bovine trabecular bone. Biomech. Model. Mechanobiol..

[47] Maquer G., Musy S.N., Wandel J., Gross T., Zysset P.K. (2015). Bone volume fraction and fabric anisotropy are better determinants of trabecular bone stiffness than other morphological variables. J. Bone Min. Res..

[48] Mathur V.P., Atif M., Duggal I., Tewari N., Duggal R., Chawla A. (2022). Reporting guidelines for in-silico studies using finite element analysis in medicine (RIFEM). Comput. Methods Prog. Biomed..

