# Supplementary material for: Mechanostat-Informed Strain Mapping of Osseodensification-Inspired Peri-Implant Densification Versus Conventional Drilling in Osteoporotic-like Low-Density Cancellous Bone: A 3D Static Linear Finite Element Analysis
Source: J Funct Biomater. 2026 Mar 18;17(3):149. doi: 10.3390/jfb17030149 (PMC13028116; doi:10.3390/jfb17030149)
Supplement: Supplementary file 1 [file jfb-17-00149-s001.zip › jfb-4186338-supplementary.pdf]

SUPPLEMENTARY TABLES

Supplementary Table S1. Mesh convergence results for high-tail peri-implant strain (Top-10 nodal mean ε_{eq}) in the crestal cortical ROI (0.5/0.3/0.1 mm).

Mesh size (mm)	Case	Nodes	Elements	Load	ROI	ε_{eq} (Top-10 mean, με)	Δ% vs previous
**0.5**	OD	242045	136930	Oblique	crestal cortical	2490	–
**0.3**	OD	495866	280362	Oblique	crestal cortical	2894	16.22
**0.1**	OD	3504209	1993628	Oblique	crestal cortical	3040	5.04

Supplementary Table S2. Component-wise mesh statistics (nodes and elements) for CD and OD models.

Models	Total		Cortical		Cancellous		Osseodensification Zone		Implant		Abutment		Screw		Crown		PTFE (Teflon) plug	
	Nodes	Elements	Nodes	Elements	Nodes	Elements	Nodes	Elements	Nodes	Elements	Nodes	Elements	Nodes	Elements	Nodes	Elements	Nodes	Elements
**CD**	2333784	1321442	873855	513201	555975	328470	-	-	254354	150263	222362	129993	58473	33398	222629	131273	146136	34844
**OD**	3504209	1993628	873850	513461	606879	356882	1120124	644296	254564	150388	222573	129836	57904	32925	222179	130996	146136	34844

Mesh statistics are reported per component to improve reproducibility. Osseodensification zone is present only in the OD model. Counts correspond to the final analysis mesh (0.1mm).
